# Molecular Mechanisms Underlying Muscle Wasting in Huntington’s Disease

**DOI:** 10.3390/ijms21218314

**Published:** 2020-11-05

**Authors:** Manuela Bozzi, Francesca Sciandra

**Affiliations:** 1 Dipartimento Universitario di Scienze Biotecnologiche di Base, Cliniche Intensivologiche e Perioperatorie, Sezione di Biochimica e Biochimica Clinica, Università Cattolica del Sacro Cuore di Roma, Largo F. Vito 1, 00168 Roma, Italy; 2 Istituto di Scienze e Tecnologie Chimiche “Giulio Natta”– SCITEC Sede di Roma, Largo F. Vito 1, 00168 Roma, Italy; francesca.sciandra@cnr.it

**Keywords:** Huntington disease, skeletal muscle, muscle atrophy, protein aggregates

## Abstract

Huntington’s disease (HD) is an autosomal dominant neurodegenerative disorder caused by pathogenic expansions of the triplet cytosine-adenosine-guanosine (CAG) within the Huntingtin gene. These expansions lead to a prolongation of the poly-glutamine stretch at the N-terminus of Huntingtin causing protein misfolding and aggregation. Huntingtin and its pathological variants are widely expressed, but the central nervous system is mainly affected, as proved by the wide spectrum of neurological symptoms, including behavioral anomalies, cognitive decline and motor disorders. Other hallmarks of HD are loss of body weight and muscle atrophy. This review highlights some key elements that likely provide a major contribution to muscle atrophy, namely, alteration of the transcriptional processes, mitochondrial dysfunction, which is strictly correlated to loss of energy homeostasis, inflammation, apoptosis and defects in the processes responsible for the protein quality control. The improvement of muscular symptoms has proven to slow the disease progression and extend the life span of animal models of HD, underlining the importance of a deep comprehension of the molecular mechanisms driving deterioration of muscular tissue.

## 1. Introduction

Huntington’s disease (HD) is a neurodegenerative pathology, caused by defects of the IT-15 gene encoding Huntingtin protein, a large 348 kDa protein predicted to form an elongated and flexible superhelix [1]. At its N-terminus, Huntingtin harbors an expandable poly-glutamines (polyQ) stretch, starting at amino acid 18, followed by a proline-rich domain. Huntingtin is conserved between *Drosophila* and mammals, but the number of glutamines increases with the evolution, with the longest known polyQ tract found in humans [2]. The proline-rich domain is found only in mammalian Huntingtin and it is crucial for the interactions with proteins containing homology region 3 (SH3) or tryptophane (WW) domains [2]. All Huntingtin orthologs contain HEAT repeats located downstream of the proline-rich domain. HEAT repeats (named according to four proteins in which they were first detected: Huntingtin, Elongation factor 3, regulatory A subunit of protein phosphatase 2A and TOR1) are formed by 40 amino acids that occur multiple times along the length of the protein and mediate protein-protein interaction [2]. Huntingtin is essential for murine embryogenesis [3,4,5] and interacts with an ever growing number of protein partners (to date they are more than 400) [6,7], being involved in many biological functions, including regulation of gene transcription, RNA splicing, protein degradation and vesicle transport [8].

HD is due to expansion of the triplet cytosine-adenosine-guanosine (CAG) within the exon 1 of the IT-15 gene generating an aberrant prolongation of the polyQ stretch [9]. Many studies reported that the CAG repeats length is inversely related to the age of HD onset [10,11]. Healthy subjects have fewer than 35 CAG repeats, with a number of CAG repeats usually comprised between 17 and 20 [12]; individuals with a number of CAG repeats comprised between 36 and 40 manifest the first symptoms in the fourth decade of life, while CAG repeats greater than 60 are associated to juvenile forms of HD [13]. The abnormal length of the polyQ stretch causes protein misfolding and aggregation, leading to alterations in the many protein-protein interaction networks in which Huntingtin is involved and a toxic gain-of-function [14,15]. Whether the toxicity of mutant Huntingtin is due to its aggregates or its soluble oligomers remains a matter of debate, since it has been proposed that mutant Huntingtin aggregates play a protective role, by removing the soluble oligomeric forms that represent the toxic species [16]. In any case, the formation of mutant Huntingtin containing aggregates is correlated with disease progression. In addition, mutant Huntingtin is particularly exposed to the activity of many proteases that generate proteolytic fragments with increased toxicity [17,18,19,20]. The enhanced proteolysis observed in HD is crucial for the pathogenesis and leads to the generation and accumulation of intracellular small toxic N-terminal fragments containing the polyQ stretch [21,22].

Typical symptoms of HD include psychiatric disorders, behavioral anomalies, cognitive decline and motor dysfunction, such as involuntary muscle contractions that cause irregular jerky movements (chorea), slow movements (bradykinesia), postural abnormalities (dystonia) and muscle rigidity [13,23,24].

When HD was identified, it was thought to be a neuronal pathology involving the central nervous system, but it soon became evident that it involves also peripheral tissues, although at different extents. Indeed, Huntingtin is widely expressed in neuronal cells, but also in many peripheral tissues, like heart, skeletal muscle, kidney, and liver, where its abundancy is comparable to that of the brain [25,26,27,28,29,30]. Dysfunction of peripheral cells also occurs when they are isolated in vitro suggesting that the pathogenesis of HD in peripheral tissues may be independent of the central nervous system. In fact, it has been shown that the same pathways that lead to neurodegeneration are also active in myoblast and blood cells cultures from HD patients and the peripheral manifestations of HD are thought to be a contributor to its morbidity and mortality [31,32]. Among peripheral tissues, skeletal muscle is heavily involved, as can be deduced by relevant clinical evidences displayed by HD patients and animal models of HD (Table 1) that experience muscular weakness [33,34] and progressive muscular wasting [35,36], which are not necessarily associated with alterations in brain functions [37,38].

**Figure 1:**
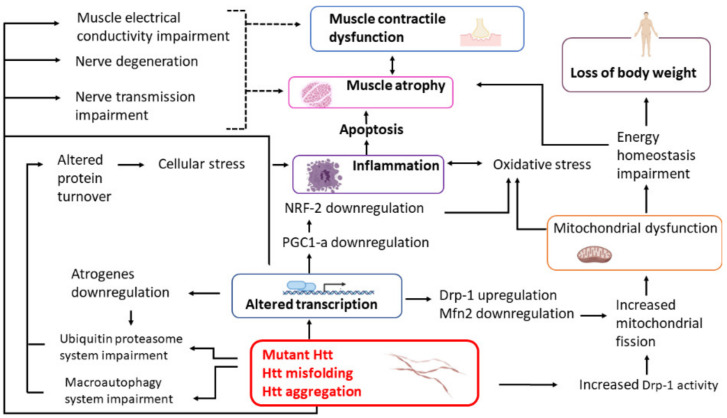
The scheme represents the main mechanisms contributing to muscle weakness and atrophy in HD and their interconnections. Dotted lines represent putative connections. **Htt**, Huntingtin. Created with BioRender.com.

This review is focused on the current knowledge of the molecular events leading to skeletal muscle wasting in HD patients with the aim of facilitating future research on the identification of novel biomarkers of the disease as well as new therapeutic strategies. The synoptic diagram reported in Figure 1 summarizes the main topics of this review.

Table 1 is intended for non-specialized readers and schematically describes the animal models mentioned in this review. In general, transgenic mice expressing truncated forms of the mutant Huntingtin have earlier and more severe phenotypes than mice expressing the full-length mutant Huntingtin. They also exhibit nuclear aggregates at precocious stages of disease. In contrast, animal models expressing full-length mutant Huntingtin display formation of cytoplasmic aggregates of Huntingtin, whereas the nuclear localization or aggregation occurs only at more advanced stages of disease [39,40,41,42].

## 2. Loss of Body Weight and Body Mass Index

Loss of body weight and body mass index is a well known hallmark of HD in early and late HD patients [57,58,59,60], pre-symptomatic HD adult [61] and children [62] patients, although calories uptake remains unaltered. These symptoms can be observed also in animal models of the disease [43,63,64,65,66,67,68,69], where a weight loss of all organs was measured in R6/2 and HdhQ150/Q150 mice [28,70]. Interestingly, a study carried out on a large cohort of HD patients showed that a high body mass index was associated with a slower rate of disease progression and that this association was independent of the length of the CAG triplets and disease stage [71]. In contrast, an analysis performed on 18 pre-symptomatic children, carrying the typical genotype of juvenile HD, revealed that, in the contest of a more severe genetic aberration, the body weight was negatively correlated to the number of CAG repeats, with the more pronounced body weight loss associated to a larger CAG repeats number [72]. The strong reduction of the body weight is probably a consequence of the skeletal muscle atrophy, since muscle mass accounts for about 40% of the total body weight [63,73]. The molecular mechanisms underlying the loss of body weight and muscle mass in HD are not completely understood, but, certainly, dysregulation of the metabolism represents an important contribute. Indeed, the levels of mRNA transcripts encoding for metabolic enzymes, such as creatine kinase, enolase and aldolase, and for proteins involved in signal transduction pathways, such as phosphatidylinositol glycan and cAMP-specific phosphodiesterase [29] and the mRNA and protein levels of the glycolytic enzyme glucose-6-phosphate dehydrogenase [65] were decreased in muscles of R6/2 mice in comparison with age-matched controls. Interestingly, in muscles of R6/2 and Hdh150Q mice and in HD patients, many changes in the expression profile overlapped with that showed by a healthy fasting group of individuals, used as a reference; for example, down-regulation of genes encoding for glycolytic enzymes was noted, although at a greater extent in R6/2 mice [74]. In physiological conditions and in a context of energy deprivation with reduced ATP/AMP ratio and reduced levels of phosphocreatine, AMP-activated protein kinase (AMPK) pathway is activated in order to stimulate glucose transport, fatty acid oxidation and mitochondrial biogenesis [75]. In muscles of NLS-N171-82Q HD mice, mRNA and protein levels of AMPK and its activity were reduced in comparison with control animals not only under basal conditions, but even in conditions of energy deprivation, chemically induced by treating animals with the catabolic stressor β-guanidinopropionic acid [76], outlining the inability of the diseased animals to maintain energy homeostasis. It is worth noting that the reduced glycolytic flux from exogenous glucose and the reduced oxidation of glucose noted in the extensor digitorum longus muscle of R6/2 and HdhQ150 mice was not revealed in the tibialis anterior muscles, suggesting that the metabolic alterations occurring in HD do not involve the different muscle types at the same extent [73]. This might reflect different muscle types composition featuring different muscle types. Indeed, each muscle fiber type displays a specific oxidative potential (see Paragraph 8).

## 3. Physiology of HD Skeletal Muscle

In 2007, Kosinski and colleagues reported a case of a pre-symptomatic HD marathon runner who developed the first signs of muscle pain, fatigue and mild myopathy years before the onset of typical HD symptoms. At more advanced stages of the disease, an analysis of muscle biopsy of this patient displayed isolated degenerated muscle fibers with an increased number of central nuclei and mild increase of glycogen deposits between mitochondria [33]. This observation suggests that muscle deficit can be considered an event independent of neuronal degeneration. HD patients exhibited a drastic reduction of lower limb muscle strength to about half of the strength of healthy age and sex matched control [24,34]. A pronounced reduction in muscular strength was observed also in animal models of HD, such as R6/2 mice [77]. In particular, in R6/2 mice at 12–14 weeks of age, the extensor digitorum longus and the tibialis anterior, two fast twitch muscles that contract and relax rapidly, exhibited a reduced maximum twitch and tetanic force, accompanied by a remarkable and progressive loss of motor units. The impairment of contractile muscle functions occurred in a context of generalized loss of muscle mass and atrophy [68,73]. In contrast, in 12 months old BACHD mice, no changes in the maximum muscular force were revealed, although a reduction in the cross-sectional area of muscle fibers from tibialis anterior was measured [51,77,78,79]. This discrepancy could be attributed to different stages of disease progression of the animal models used for muscle strength tests. A possible contribution to the reduced muscular strength may be provided by a reduced Ca^2+^ reuptake capability, expressed by the sarcoplasmic reticulum and mediated by the SERCA calcium pump, and by a reduced Ca^2+^ flux, released from the sarcoplasmic reticulum via the ryanodine receptor in response to an action potential, found in the interosseous muscle fibers of R6/2 mice [80]. Indeed, it was hypothesized that a permanently high myoplasmic Ca^2+^ concentration could activate Ca^2+^dependent proteases, such as calpains [81]. Proteolysis of the connecting protein junctophilin could uncouple the transverse tubules from the sarcoplasmic reticulum, resulting in the impairment of voltage-activated Ca^2+^ release [80].

## 4. Morphology of HD Skeletal Muscle and Protein Aggregates

An analysis performed post-mortem on muscular tissues extracted from HD patients evidenced the presence of granular deposits in muscle fibers characterized by centrally localized nuclei [82]. The results obtained on human tissues are similar to those found in HD animal models. In a histological analysis performed on R6/2 and R6/1 mice, the appearance of granular inclusions, positive to the ubiquitin and Huntingtin staining and located inside the nuclei of muscle fibers, was revealed starting at 6 weeks of age and constantly increasing with age [28,63]. The age dependent increase of mutant Huntingtin inclusions in muscle tissue was inversely related to the decrease of the soluble mutant Huntingtin [83]. Further studies highlighted that the Huntingtin positive aggregates were mainly located at the perinuclear zone, rather than inside the nucleus or the cytosol, whereas the amount of the soluble form of mutant Huntingtin was higher in the cytosol/mitochondrial fraction than in the nucleus [84]. Huntingtin positive aggregates were also found at the periphery of muscle fibers that also showed the presence of non-muscle nuclei, indicative of inflammatory cells infiltrations [85]. Discrepancies in the subcellular localization of Huntingtin aggregates found in different studies are probably due to different antibodies used for their immunodetection. Indeed, different antibodies directed against the exon 1 of Huntingtin are available that display different affinities toward the soluble or multiple oligomeric forms in which Huntingtin may exist. Nuclear inclusions positive to the Huntingtin antibody were also found in myocytes from HdhQ150/Q150 knock-in mice, at 22 months of age [70,73]. The increase of aggregates, both in number and in size, always paralleled the development of muscle atrophy. In R6/2 mice, the atrophic phenotype affected many muscle types, including flexor digitorum brevis, quadriceps, soleus, interosseous and femoris muscles (this latter also showed infiltrations of non-muscular tissue). All these muscles displayed an increased variability of muscle fibers diameters with a decreased average value compared to that of wild-type animals [28,63,80,86]. Muscle atrophy was accompanied by a significant weight loss of the animals and was often found to be independent of denervation. Of note, atrophy was extended homogenously to all types of muscle fibers [28,63]. However, only at 15 months of age R6/1 mice displayed clear signs of muscle degeneration, characterized by increased mitochondrial size with normal cristae, decreased number of post-synaptic folds and endplate associated fibrous structures and less articulated motor endplates [28]. An analysis of HdhQ150/Q150 mice muscles also reported muscle fibers with reduced diameter and central nuclei. The presence of split fibers was also noticed [70,73]. Anomalies of the muscle fibers morphology was observed also in 12 months old BACHD mice, which undergo a slower disease progression, with respect to R6/2 mice. Indeed, fibers of tibialis anterior muscle displayed a marked disorganization of sarcomeres that lost their typical alignment, an intrusion of the sarcoplasmic reticulum onto the myofibrils zone and a remarkable amount of intermyofibrillar glycogen [79]. From all these studies emerges the notion that a correlation could be established between muscular atrophy and the presence of mutant Huntingtin aggregates, since these two phenomena often coexist. A further hint concerning the toxicity of the mutant Huntingtin aggregates for muscle tissue has been provided by a recent study reporting downregulation of target genes of the transcriptional regulator Myocyte Enhancer Factor 2 (MEF2), a regulator of myofiber homeostasis, but not of the MEF2 gene transcript. The findings that MEF2 co-localized with p62, which is a marker of inclusions, within the muscular fibers of R6/2 mice, and that the recombinant protein GFP-74Q Huntingtin was co-immunoprecipitated with MEF2, raised the hypothesis that the inclusion of MEF2 within the polyQ-Huntingtin aggregates impairs its function of transcriptional regulator, contributing to the muscular atrophy found in HD [87].

## 5. Does Mutant Huntingtin Affect Differentiation of Skeletal Muscle Cells?

A correct differentiation process is necessary to activate a proper regeneration pathway to replace damaged muscular fibers. In order to investigate whether the differentiation process is altered in HD skeletal muscle, some studies have been focused on the analysis of the Huntingtin expression pattern and its aggregation during myoblast differentiation. An immunofluorescence analysis performed on primary myoblasts, extracted from pre-symptomatic and symptomatic HD patients, revealed a homogenously diffused cytosolic staining for Huntingtin, in a context of irregular cellular shape, cellular fragmentation and cytoplasmic shrinkage. In the control and HD differentiated myotubes, the Huntingtin staining was more intense, but Huntingtin immunoreactive protein inclusions were found only in the cytosol of HD myotubes; a small fraction of such inclusions was also ubiquitin positive [31]. The Huntingtin expression pattern found in human cells was similar to that observed in muscle cell cultures from 12 weeks old R6/2 and control mice that started to express Huntingtin only 1 week after the switch from the growth to the differentiation medium. The intense Huntingtin expression was always accompanied by the expression of desmin, a marker of myoblast differentiation, whereas very low levels of Huntingtin expression were found in desmin negative cells, indicating that Huntingtin is almost exclusively expressed during myoblast differentiation. In control and HD cells, Huntingtin expression dropped remarkably at 4 weeks after differentiation reaching the basal levels found in desmin negative cells. At 6 weeks after differentiation, HD cells displayed ubiquitin positive Huntingtin inclusions inside the nucleus, although at that time staining of soluble Huntingtin was strongly reduced in healthy and HD cells. Cytosolic inclusions that are positive to ubiquitin and Huntingtin could be observed only in HD desmin negative cells [88]. It is not clear how the expression of mutant Huntingtin affects myoblast differentiation, since conflicting data are reported. In human cells, whereas the myoblast proliferation showed no differences between control and HD cells, the differentiation process from myoblasts to myotubes was slower in HD [31]. On the other hand, another study performed on muscle cells extracted from HD patients showed anomalies in morphology and pattern of growth for both, myoblasts and myotubes [76]. In muscle cells from 12 weeks old R6/2 mice, the differentiation process from myoblasts to myotubes was faster in HD than WT mice [88]. Unfortunately, transcriptomic analysis did not contribute to clarify this point. Indeed, a microarray analysis, carried out on 12 weeks old R6/2 mice, reported a reduction of the mRNA levels of genes encoding for proteins typical of the terminal muscle differentiation, such as myoD, actin, myosin light chain and troponin [29]. In contrast, a transcriptomic study performed on isogenic HD human embryonic stem cell lines, induced to differentiate in muscle cells and carrying varying CAG repeat lengths in the first exon of Huntingtin, did not find any differences in the transcription levels of markers corresponding to the different stages of muscle differentiation [89].

## 6. Activation of Cellular Stress Response

### 6.1. Protein Synthesis and Degradation 

The reasons for muscle atrophy that characterizes the HD phenotype are not completely understood. Loss of muscle mass occurs when the equilibrium between protein synthesis and protein degradation is altered. The mammalian target of rapamycin (mTOR) signaling pathway plays a key role in the protein synthesis and it is stimulated by nutrients, especially leucine, and hormones and growth factors, such as insulin and insulin-like growth factor 1 (IGF-1) [90]. In physiological conditions, the protein turnover is assured by the ATP-dependent ubiquitin proteasome and autophagy–lysosome systems that represent the two main proteolytic pathways within skeletal muscle. In the ubiquitin proteasome system, damaged or unnecessary proteins are covalently bound to poly-ubiquitin chains and delivered to the proteasome, a large protein complex (26S) responsible for the proteolysis. Damaged proteins can be removed also by the lysosomal proteolytic system through a process called macroautophagy. Macroautophagy is a type of autophagy in which whole regions of cytosol containing macromolecules and organelles are sequestered in double-membrane vesicles known as autophagosomes to permit their degradation after fusion with lysosomes. Calcium-dependent calpain and caspase systems are also involved in protein degradation, albeit at a minor extent [91]. All these catabolic processes assure the correct removal of damaged proteins in physiological conditions, but their excessive activity leads to muscle atrophy [92,93,94]. On the other hand, decline of the macroautophagy process occurring with age is one of the main causes of the age-dependent reduction of muscle mass and strength, called sarcopenia [95]. The relevance of defective macroautophagy in sarcopenia is supported by finding that promoting macroautophagy prevented loss of muscle mass and strength, by increasing the selective removal of misfolded proteins and damaged organelles [96,97,98]. In HD, mutant Huntingtin forming soluble oligomers and/or aggregates should elicit activation of proteolytic events aimed at removing these toxic species. As a matter of fact, studies based on oligonucleotide microarrays performed on muscles extracted from animal models of HD and HD patients reported increased mRNA levels of genes that encode chaperones, heat shock proteins, proteasomal-subunits, ubiquitin-conjugating enzymes and multiple DNA-repair enzymes, supporting the hypothesis of a cellular reaction to the toxicity related to Huntingtin aggregates [29,74]. In addition, the mRNA abundancy of Foxo-3 (Fork head box O3), a transcription factor that regulates muscle mass and when overexpressed induces muscle atrophy [99,100,101], was markedly increased, although those of muscle creatinine kinase (Mck), a marker of muscle damage, was decreased in tibialis anterior, extensor digitorum longus and gastrocnemius/plantaris complex muscles from the R6/2 and HdhQ150 knock-in mice, compared to control animals [73]. On the other hand, the mRNA levels of the atrogenes MuRF1 and MAFbx, that play a key role in the ATP-dependent ubiquitin proteasome system, were reduced after a prolonged period of fasting in R6/2 mice in comparison to control mice. This indicates that protein degradation mediated by the ATP-dependent ubiquitin proteasome system was not increased in muscles of R6/2 mice, not even in starvation, a condition that is known to promote protein degradation, in order to recycle amino acids. This was confirmed by observing a slight reduction in the 3-methylhistidine/creatinine ratio measured in the urine of R6/2 mice with respect to the control mice [65]. Indeed, since 3-methylhistidine is an amino acid contained only in the α-actin and myosin, two proteins highly expressed in muscular tissue, it is considered a marker of the ATP-dependent ubiquitin proteasome system, whereas the creatinine, a degradation product of the creatine phosphate, is related to the muscle mass. Both these metabolites are excreted in the urine. The impairment of the ubiquitin proteasome system in HD was found also in the central nervous system. For example, in neurons, 26S proteasome was unable to digest the polyQ stretch that obstructed its passage impairing its chymotrypsin and caspase like activities [102,103]. As far as macroautophagy is concerned, in gastrocnemius muscle of R6/2 and control mice, it was observed an overexpression of the microtubule-associated protein light chain LC3, which is crucial for the expansion of autophagosomes, both in its cytosolic form (LC3B-I) and in the membrane bound form (LC3B-II), although the LC3B-I/LC3B-II ratio was not different. In addition, Beclin-1, which is associated with multimeric complex of macroautophagy regulatory proteins, the atg7 protein, which is responsible for the conjugation of atg12 to atg5 and LC3 to phosphatidylethanolamine, and atg5, a protein involved in the expansion of the autophagosome precursor and its completion through an ubiquitin-like conjugation system, were upregulated in R6/2 mice [65]. Upregulation of genes involved in macroautophagy in R6/2 mice probably represents a compensatory adaptation to the disease, but it does not prove an augmented autophagic flux within the skeletal muscle. It is worth noting that, in physiologic conditions, Huntingtin itself promotes autophagosome formation, by serving a role of protein scaffold through its C-terminus. The interaction between the Huntingtin N- and C-termini modulates this function [104,105,106], but the multiple proteolytic events occurring preferentially on the mutant Huntingtin disrupt this interaction, impairing the whole macroautophagy process in central nervous system [107]. Indeed, mutant Huntingtin alters autophagosomal dynamics and interferes with initiation of autophagy [108]. Surprisingly, mTOR activity, which stimulates the protein synthesis in skeletal muscle, was increased in muscles of R6/2 mice when compared to wild-type animals; indeed, it was reported an upregulation of the insulin/IGF receptor-PI3K-Akt signal transduction pathway, which leads to the mTOR activation, and a downregulation of the AMPK pathway that inhibits mTOR activity [65]. These data are quite unexpected in a context of general muscular atrophy (see Paragraph 4). Nevertheless, it should be considered that excessive mTOR activation may trigger deleterious effects. This is supported by findings that mice knocked-out for TSC1, an mTOR inhibitor, developed late-onset myopathy due to impaired autophagy [109]. In addition, it has been revealed that upregulation of mTOR activity reduced global protein synthesis by promoting eIF4F complex formation [110] and triggered a FoxO-induced cascade of catabolic events by inactivating Akt [111], thus contributing to muscular atrophy induced by denervation and immobilization [110,111]. However, a recent study performed on R6/2 mice, showed a reduced expression of IGF-1 in muscle, where IGF-1 plays an autocrine/paracrine role, and liver, which synthesizes the most part of this growth factor [112], consistent with the reduced levels of circulating IGF-1 found in plasma of R6/2 mice and HD patients [113]. Of note, the IGF-1 levels decline during aging, contributing to the sarcopenic phenotype [114]. Collectively, these data suggest that it is unlikely that muscular atrophy featuring HD may be attributed to an excessive activation of the ubiquitin-proteasome and autophagy-lysosome pathways, leading to an abnormal protein degradation that would prevail on protein synthesis. On the contrary, it can be hypothesized that muscle atrophy may be ascribed to impaired protein turnover with consequent accumulation of misfolded proteins, which provokes cellular stress.

### 6.2. Inflammation

Inflammation is a prominent feature of muscular wasting and the transcription factor NF-kB may often drive activation of atrophic program. Under basal conditions, NF-κB forms a cytosolic complex with its inhibitors, belonging to the IκB family. During inflammation, circulating cytokines, such as tumor necrosis factor α (TNFα) and interleukin-6 (IL6), induce IκB inhibitors degradation that allows NF-kB entering the nucleus, where it promotes the transcription of atrogenes involved in protein degradation. The NF-kB relevance for muscle atrophy was supported by finding that overexpression of Iκβα, a member of the IκB inhibitors family, prevented disuse atrophy by suppressing the NF-κB activity [115,116]. It is worth noting that increased abundancy of proteins involved in the NF-κB signaling pathway, such as p65/RelA, Tradd, and TRAF5, was revealed in skeletal muscle of R6/2 and full-length knock-in HdhQ175 mice [86,117]. Overexpression of NF-κB found in animal models of HD is consistent with up-regulation of its target Pax7, a marker of muscle satellite cells that replenishe damaged muscle fibers, in tibialis anterior muscle from R6/2 mice at 12 weeks of age [68]. It has been reported that persistent expression of Pax7 in healthy muscles leads to atrophy, whereas its reduction restores muscle regeneration in mice affected by cancer [118]. In addition, increased circulating levels of IL-6 and TNF-α were reported in the plasma of pre-symptomatic and HD patients, indicating that a persistent inflammatory condition is a hallmark of HD, even before the onset of the classical symptoms [119]. Remarkably, TNF-α and IL-6 have been associated with muscle loss and lower strength in a large sample of older persons [120,121]. In particular, increased levels of circulating IL-6 were shown to foster muscle atrophy and dysfunction by altering the redox balance of muscular tissue [114].

### 6.3. Apoptosis

Apoptosis is a program of cell death that contributes to a physiological homeostasis, by removing damaged cells, a process often activated in response to inflammatory cytokines. Apoptosis is a key element that could provide a major contribution to muscular atrophy observed in HD. Activation of apoptotic processes takes place in the HD skeletal muscle, as indicated by many studies reporting up-regulation of different caspases. A remarkable activity of caspases 3, 8 and 9 was measured in primary muscle cells extracted by pre-symptomatic and symptomatic HD patients [31]. Cytochrome c is a heme protein involved in the electron transport chain of the oxidative phosphorylation and is associated to the inner membrane of the mitochondria. Upon apoptotic stimuli, mitochondria release Cytochrome c into the cytosol, where induces the activation of the caspase-3 and -9 beginning the apoptotic process. In muscle of R6/2 mice, an increased cytosolic and a reduced mitochondrial concentration of Cytochrome c were measured, in comparison with healthy animals. Furthermore, the mRNA levels of genes involved in the apoptotic process, such as caspase-1, -3, -6 and -9, were increased as well as the mRNA level of Cradd, which is associated with caspase-2, the death receptor Fas (CD95/Apo1), its ligand FasL, and death domain and binding cofactor FADD. The protein counterparts of procaspase-9 and -3, Fas and FADD were also increased in R6/2 mice, and this upregulation was paralleled by an increased activity of caspase-3/7, -8, and -9 [65]. Similar experimental evidence corroborates the notion that apoptotic pathways are activated in HD. Up-regulation of genes encoding for caspases 3 and 8 was observed in skeletal muscle of R6/2 and full-length knock-in HdhQ175 mice [117]. Caspase 8 transcripts were found to be overexpressed also in tibialis anterior, extensor digitorum longus and gastrocnemius/plantaris complex muscles from the R6/2 and HdhQ150 knock-in mice [73]. Increased expression of caspase 3, caspase 8, Smad3, Traf-5 and Creb1, whose activity is related to muscle injury [122], was measured in gastrocnemius skeletal muscle from 14 weeks old R6/2 mice, compared with wild-type mice [86]. In the hindlimb muscle from 12 months old YAC128 and in 12 weeks old R6/2 mice, the caspase-6 activity was increased compared to control animals; in addition, in post-mortem muscle tissues from HD patients there was an increased fragmentation of lamin A, that is a known caspase-6 target [123]. The increased caspase-6 mRNA levels have been associated with an increased p53 transcription; interestingly, inhibition of the p53 transcriptional activity restored the normal caspase-6 mRNA levels and, at a minor extent, the activity of this enzyme. This experimental evidence supports the notion that the increased expression and activity of caspase 6 may be triggered by a transcriptional dysfunction, probably induced by mutant Huntingtin or its fragments, that leads to p53 upregulation [123]. All together, these data substantiate the hypothesis that apoptosis and inflammation may play key roles in muscle atrophy in HD.

## 7. Mitochondrial Dysfunction in HD Skeletal Muscle

### 7.1. Deficits of the Energy Metabolism

A pioneering study, based on 31P magnetic resonance spectroscopy, reported a drastic reduction of the phosphocreatine to inorganic phosphate ratio in muscles of HD patients at early stage of the disease without signs of myopathy, suggesting a defective oxidative metabolism [124]. A reduced phosphocreatine to inorganic phosphate ratio was measured also in another group of HD patients but not in pre-symptomatic individuals, while a reduced ATP/(phosphocreatine + inorganic phosphate) ratio was found in both groups of patients, although at a minor extent in the pre-symptomatic condition. Alterations of these metabolic parameters were evident in muscle at rest, during exercise and during recovery after exercise. Of note, the decreased maximal rate of ATP production, corrected for the age of patients, was correlated with the CAG repeats length in symptomatic but not in pre-symptomatic patients [125]. A prolonged phosphocreatine recovery after exercise was found also in another study carried out on symptomatic as well as pre-symptomatic HD patients, although the phosphocreatine/inorganic phosphate and the ATP/(phosphocreatine + inorganic phosphate) ratios at rest were not different with respect to healthy subjects. No correlations among oxidative function and/or motor performances and genetic parameters were evidenced [82]. However, the reduced oxidative capacity of mitochondria observed even in pre-symptomatic HD patients, who still do not show signs of muscle atrophy, suggests that energy metabolism impairment is a primary event occurring in the HD development. This notion is supported by observing that both, pre-symptomatic and symptomatic HD patients displayed a reduced anaerobic threshold, a parameter used to evaluate the exercise capacity. During the early phase of cardiopulmonary exercise, the muscle is well supplied with oxygen and the ATP necessary to support intense muscle activity is provided by oxidative phosphorylation. When the oxygen supply fails to meet the increasing energy demand of the muscle, the glycolytic pathway is activated and a shift from aerobic to anaerobic metabolism occurs, with a consequent increase in lactic acid production. This shift occurred earlier in pre-symptomatic and in symptomatic HD patients than control subjects, although only symptomatic HD patients showed reduced work capacity with respect to healthy subjects, indicating that muscle atrophy and loss of muscle mass occur during more advanced phases of HD. Interestingly, the anaerobic threshold was lower in pre-symptomatic HD patients displaying the highest number of CAG repeats, supporting the hypothesis that mutant Huntingtin interferes directly with mitochondrial respiratory chain. On the other hand, the anaerobic threshold was not related to the number of CAG repeats in symptomatic HD patients, suggesting that other elements occur later during HD progression [126]. Different animal models of HD recapitulate the pathologic characteristics exhibited by HD patients. In 26-week-old NLS-N171-82Q HD mice, reduced levels of ATP but not of phosphocreatine, creatine, ADP and AMP were found compared to wild-type animals [76]. A study carried out on extensor digitorum longus, tibialis anterior and soleus muscles from R6/2 and HdhQ150 knock-in mice evidenced a significant reduction of the phosphocreatine/creatine ratio and the entire pool of the adenine nucleotides, while a much slighter reduction of the redox status was observed [73]. 

### 7.2. Aberrations of the Mitochondrial Morphology

Mitochondria serve a crucial role in preserving metabolic homeostasis and cell survival and for this reason they are subjected to a fine quality control based on mitochondrial biogenesis, mitophagy, mitochondrial proteostasis and dynamics [127]. Mitochondria are extremely dynamic organelles that undergo fusion and fission processes. Mitochondrial fusion facilitates the exchange of components, such as metabolites, proteins and DNA between two mitochondria, in order to repair each other, while mitochondrial fission allows the segregation of damaged mitochondria from the healthy ones [128,129,130]. Mitochondrial fusion is coordinated by two proteins called mitofusins 1 and 2 (MFN1 and MFN2), embedded within the outer mitochondrial membrane, and by a protein belonging to the inner membrane called optic atrophy 1 (OPA1) [131,132]. The fission process is mediated by the GTPase dynamin-related protein-1 (Drp-1), which is recruited by adaptor proteins at specific mitochondrial sites [131,132]. The mitochondrial morphology and density and the muscle fibers integrity depend on the fusion and fission processes. This view is supported by studies showing that reducing or completely deleting the genes encoding for MFN1, MFN2 and OPA1 leads to severe mitochondrial dysfunction, muscle atrophy and myopathy [133,134,135]. Conversely both, overexpression [136,137] as well as complete [138] or partial [139,140] removal of the Drp-1 gene cause mitochondrial aberration with reduced respiration capacity, accompanied by muscle wasting and degeneration. It was also proposed that a drastic reduction of Drp-1 may compromise the neuromuscular junction integrity [140]. It has been evidenced that Drp-1 forms a more stable complex with mutant Huntingtin than with wild-type Huntingtin in human brain and lymphoblasts and in murine brain; in addition, mutant Huntingtin increases the enzymatic activity of the Drp-1, promoting an excessive mitochondria fragmentation [141,142,143]. An increased expression of Drp-1 and its phosphorylated activated form was found in quadriceps of 12 weeks old R6/2 mice, whereas the expression levels of the pro-fusion factor MFN2 were found to be reduced [84,86]. In HD, deficits in energy metabolism are accompanied by alterations of mitochondrial morphology. Indeed, an increased number of mitochondria with abnormal cristae was observed in muscle fibers of four HD patients that did not display muscle symptoms [144]. An increased number of mitochondria that were also enlarged in size was revealed in a single HD patient [33]. Derangement of the internal structure of mitochondria was related to the amount of mutant Huntingtin expressed in muscle: a comparative analysis of the mitochondria morphology, performed on primary cell cultures from deltoid muscle of HD patients, evidenced more pronounced alterations in homozygous rather than heterozygous HD patients [145]. Mitochondrial anomalies were observed also in animal model of HD. Indeed, mitochondria with irregular shape and exhibiting poor alignment were observed in muscle of NLS-N171-82Q and R6/2 mice [76,146], whereas increased mitochondrial density was reported in muscle of R6/2 mice and transgenic minipigs [147,148]. Mitochondria of BACHD mice muscle fibers contained large vacuoles, which are sign of cell degeneration enrolled by mitochondria [79]. 

### 7.3. Impairment of the Mitochondrial Electron Transport Chain

As expected, impairment in the energy metabolism and aberrations of the mitochondrial structure are mirrored by functional anomalies of the enzymes involved in the oxidative phosphorylation. Impairment of complex I was first reported in muscle of four HD patients. Interestingly, the complex I activity was inversely correlated with the CAG repeats length [144]. In a cohort of 12 HD patients, it was found a significant reduction of complexes II-III activity, which correlated with a parameter obtained by multiplying the patient age per the number of CAG repeats, whereas complex I and IV (cytochrome C oxidase), as well as the citrate synthase enzyme, displayed an activity comparable with that of healthy subjects [36]. In another study carried out on a single HD patient, normal enzyme activities for complex I and III and a significant reduction in the complex IV activity were measured, while an increased activity of the enzyme citrate synthase was revealed [33]. Primary muscle cells obtained by HD patients displayed a reduced oxygen consumption at rest also in uncoupling conditions [76]. In vastus lateralis muscle from pre-symptomatic HD patients, it was not found any impairment in any of the enzymes belonging to the mitochondrial electron transport chain [149]. The discrepancies emerging from these studies are probably due to differences in the methods employed for enzymatic activity measurements and in the types of muscle analyzed; another element contributing to contradictory results is represented by the heterogeneity of patients involved, as they displayed different stages of disease progression. However, a transcriptomic and proteomic analysis, performed on myotubes derived from isogenic HD human embryonic stem cells carrying CAG repeats of different length within the first exon of Huntingtin, confirmed that the main anomalies of the transcription/expression patterns concern genes involved in the mitochondrial respiration and ATP production [89].

Conflicting results were obtained also on animal models of HD. Indeed, no alterations of the mitochondrial electron transport chain activity were found in skeletal muscle of 12 weeks old R6/2 mice [150] or in skeletal muscle of 15 months old HdhQ111 knock-in mice [149]. On the other hand, other analysis, performed on muscles of R6/2 mice at late stages of disease progression, reported a significant reduction of the activity of the sole complex IV [147] or of both, complex I and IV [84]. This is in accordance with a reduced succinyl dehydrogenase staining, which correlates with mitochondrial activity, found not only in R6/2, but also in NLS-N171-82Q mice, compared to control animals [76,146]. More recently, a significant reduction in the activity of complex I, complex II, complex IV and citrate synthase was revealed in transgenic minipigs, expressing the N-terminal portion of mutant human Huntingtin, at 48 months of age, when the muscle performances undergo an observable decline [148].

Interestingly, a complete inhibition of the complex I–dependent respiration was observed in isolated mitochondria, extracted from muscles of R6/2 mice, treated with Ca^2+^ concentrations that slightly affect respiration in mitochondria from wild-type animals. This Ca^2+^ induced effect was accompanied by a higher extent of uncoupled oxidative phosphorylation, indicating an overall greater mitochondrial susceptibility to Ca^2+^ of skeletal muscle of R6/2 mice with respect to control animals [147]. 

### 7.4. Role of PGC1-α in the Mitochondrial Anomalies and Abnormal Distribution of Fiber Types 

Peroxisome proliferator–activated receptor gamma coactivator-1 (PGC-1α) is a transcriptional cofactor that coactivates many transcription factors, such as peroxisome proliferator-activated receptors (PPARs), estrogen related receptors (ERRs) and nuclear respiratory factors (NRFs), to promote transcription of genes involved in the oxidative phosphorylation and to modulate the distribution among slow-twitch oxidative and fast-twitch glycolytic fibers in the skeletal muscle; moreover PGC-1α induces a number of nuclear-encoded mitochondrial genes and simultaneously regulates the replication and transcription of the mitochondrial genome, stimulating mitochondrial biogenesis. At the posttranslational level, PGC-1α is activated by AMP-kinase (AMPK) and sirtuin 1. It is a well-established notion that PGC-1α plays a key role in modulating the adaptation of skeletal muscle to physical activity. Indeed, transgenic mice, characterized by a muscle specific PGC-1α over-expression, displayed more oxidative myofibers, increased mitochondrial activity, and enhanced endurance performance [151]. In contrast, mice, in which the PGC-1α gene has been abrogated, exhibited mitochondrial dysfunction, motor disorders characterized by hyperkinetic movements and striatal degeneration, all features also observed in HD [152,153]. As a matter of fact, the mRNA levels of PGC-1α and some of its target genes were downregulated, in muscle tissues and primary myoblast extracted from HD patients, as well as in animal models of HD, such as NLS-N171-82Q [76] and R6/2 mice [146]. It should be noted that, down-regulation of PGC-1α and its target genes occurred in a context of general transcriptional dysregulation. Indeed, by analyzing the transcriptome of quadriceps and tibialis anterior muscles in 12 weeks old R6/2 mice, it was found that about 50% of all genes were dysregulated in these muscles [68].

PGC-1α downregulation probably provides an important contribution to the mitochondrial dysfunctions described above. Nevertheless, the abnormally low abundancy of PGC-1α influences other aspects of muscle function in HD. Among the target genes of PGC-1α, NRF-2 plays a crucial role, being a master gene that coordinates cellular response to the oxidative stress. NRF-2 down-regulation, found in gastrocnemius skeletal muscle of R6/2 mice, suggests that skeletal muscle is unable to activate adequate defense processes against oxidative stress; this certainly represents a further contribution to muscle waste in HD [86].

The strong downregulation of PGC-1α impairs adaptation of skeletal muscle to chemically induced energy deprivation, as can be observed by treating NLS-N171-82Q mice with β-guanidinopropionic acid, a creatine analogue, which reduces the intracellular phosphocreatine and creatine levels. Administration of β-guanidinopropionic acid to mice activates biochemical pathways that resemble that activated during muscle adaptation to endurance exercise training. Indeed, β-guanidinopropionic acid treatment increased the number of mitochondria and stimulated the expression of AMPK, PGC-1α, PGC-1β, Tfam, COX-IV, PPARα, PPARδ, Cytochrome C and cAMP response element binding protein (CREB) that are involved in oxidative phosphorylation, electron transport chain and muscle function in wild-type mice, whereas had no effect on HD mice [76]. In muscles of wild-type mice there were a greater type I fibers/type II fibers ratio and an enhanced number of mitochondria with respect to HD and β-guanidinopropionic acid exacerbated these differences, further reducing the amounts of mitochondria and type I in comparison with type II muscle fibers in HD mice. Overexpressing PGC-1α in HD mice restored the muscle ability to adapt to energy deprivation [76].

## 8. Muscle Fiber Type Transition

The studies of Chaturvedi and Johri’s groups revealed a correlation between PGC-1α down-regulation and transition from slow oxidative to fast glycolytic muscle fibers [76,146]. Nevertheless, other studies, performed on human HD patients and animal models of HD, reported an opposite transition from fast glycolytic to slow oxidative muscle fibers. These findings suggest that still unknown factors, beside PGC-1α downregulation, must affect muscle fibers distribution in HD. 

Muscles are composed by two main fiber types, 1 and 2. Type 1 fibers are characterized by high resistance to fatigue, are rich in mitochondria and undergo an oxidative metabolism. They display a slow time-to-peak tension and a slow maximal shortening velocity. Type 2 fibers have a reduced resistance to fatigue and undergo a glycolytic metabolism, having a faster time-to peak tensions [154,155]. A further subdivision of the two fiber types is commonly based on the expression of specific myosin isoforms. Myosin (My) is a myofibrillar protein specifically expressed in muscle fibers and is composed by three subunits, one heavy (H) and two light (L) chains. There are four MyH chain isoforms, I (Myh7), IIa (Myh2), IIx/d (Myh1) and IIb (Myh4), and five MyL chain isoforms, 1f, 2f, 3f, 1s and 2s, divided in essential (1 and 3) and regulatory (2), with the letters f and s referring to fast glycolityc (f) and slow oxidative (s). The four muscle fiber types, I, IIa, IIx/d and IIb express the respective heavy chain isoforms, with I and IIa representing slow oxidative fibers and IIx/d and IIb fast glycolytic fibers (type I has the highest oxidative capacity and IIa, IIx/d, and IIb have high, intermediate, and low oxidative capacity, respectively, with a corresponding increase in the contraction velocity and a reduction in resistance to fatigue) [155,156]. In healthy individuals, physical exercise leads to a conversion from type IIx/d or IIb to the more oxidative type IIa [157].

A microarrays study compared the expression patterns of muscle obtained from 11 and 15 weeks old R6/2 mice, 6 months old HdhQ150 homozygous knock-in mice and HD patients, evidencing similar changes within these three groups of subjects compared to their healthy counterparts. Among these changes, a significant reduction of genes specifically expressed in fast muscle fibers and an up-regulation of genes specifically expressed in slow muscle fibers were observed [74]. Further transcriptomic studies, performed on muscles mainly constituted be fast glycolytic fibers, such as tibialis anterior, extensor digitorum longus and the gastrocnemius/plantaris complex muscles confirmed a significant shift of the fast MyHC isoforms toward slower MyHC isoforms, in R6/2, HdhQ150, HdhQ175 [69,73,117] and BACHD [79] mice. In addition, in R6/2 and HdhQ175 adult mice, the altered distribution among fast and slow MyHC isoforms was accompanied by an increase of a neonatal isoform of MyHC, that is expressed exclusively during development in wild-type animals [69]. In peroneal muscle of wild-type animals, about one half of the total number of muscle fibers is represented by fibers type I, while in R6/2 mice, muscle fibers type I accounted for about 80% of total muscle fibers [63]. An analysis of the protein contents of the different MyHC and MyLC isoforms, performed on the soleus muscle, which expresses mainly MyHCI and MyHCIIa and a small percentage of isoform MyHCIIx in wild-type mice, exhibited a shift from the MyHCIIa to the MyHCI isoform, but this transition involved only R6/2 male animals. A transition from the MyHCIIb to the MyHCIIx isoform was noted in the extensor digitorum longus muscle, which is normally characterized by fast glycolytic fibers. This phenomenon was more pronounced in females. It is noteworthy that also the expression pattern of the MyLC isoforms was altered in the extensor digitorum longus muscle, with a transition from the MyLC3f to the MyLC1f isoform. Interestingly, a reduced contractile force was measured for the extensor digitorum longus muscle, but not for the soleus. This suggests that the contractile functions mainly depend on the altered distribution of the fast MyLC isoforms and on the shift of the fast MyHC isoforms towards slower types, whereas an altered distribution among the slow MyHC isoforms (as those observed in the soleus muscle) has little effect on the muscle performances [158]. An altered distribution of the MyHL isoforms was revealed also comparing the interosseous muscles, mainly composed of IIa fibers, of R6/2 and wild-type mice, whereas no changes in the expression pattern of the MyHC isoforms were observed [80]. The mechanisms that drive the shift from glycolytic to oxidative muscle fibers are largely unknown, but it could be hypothesized that the reduced glycolytic metabolism observed in HD animal models [65,73,74] could have a major negative impact on the glycolytic fast rather than the oxidative slow muscle fibers. Moreover, it has been proposed that the reduced amounts of mitochondria contained in fast glycolytic muscle fibers could render them more susceptible to mitochondrial dysfunction [158]. Nerve degradation could be another key element for the altered distribution of muscle fiber types observed in HD (see Paragraph 9). 

## 9. Defects of Motoneurons

It is still matter of debate if muscular atrophy observed in HD patients and in animal models of HD is a primary muscular defect, due to high expression levels of mutant Huntingtin in the skeletal muscle, or it is a consequence of nerve degeneration. 

An analysis of the quadriceps of 14-weeks old R6/2 and 15 months old R6/1 mice did not evidence anomalies in the motor endplate distribution, nor alterations of the muscle fiber morphology, such as fibrosis, necrosis or infiltrations of non-muscular tissues [28]. Ribchester and colleagues found that at late stages of the disease R6/2 mice displayed a pronounced muscle atrophy, although there were no signs of myopathy, such as regeneration, inflammation or fibrosis, nor anomalies in nerve terminal morphology and physiology if not in a small percentage of neuromuscular junctions. Indeed, anomalies in the end plates and motor unit responsiveness were found only in 5% of the elements analyzed in different muscles and only at 18 weeks of age of the mice, i.e., at the very terminal stage of disease progression, the amount of degenerated motor units increased until an involvement of about 20% of the elements analyzed. The only phenomenon that could justify muscle atrophy was an impairment of neuromuscular junction transmission [63]. In the absence of any signs of terminal nerve degeneration, it was observed an enhanced neurotransmitter release by the neuromuscular junctions of the levator auris longus from R6/1 mice with respect to control animals. Interestingly, neuromuscular junctions of R6/1 but not wild-type mice expressed high levels of Huntingtin, although not homogenously distributed among all the neuromuscular junctions, and Huntingtin expression was accompanied by an increased expression of three synaptic vesicle proteins, namely, synaptobrevin 1/VAMP1, synaptobrevin 2/VAMP2 and CSP-α, and of the SNARE protein, SNAP-2, compared to wild-type animals [159]. This latter result is in accordance with other studies showing an interaction between the mutant Huntingtin and cytoskeletal synaptic vesicles proteins that serve crucial roles for the integrity of neuromuscular junctions and for exocytosis and endocytosis of synaptic vesicles at the nerve terminals [160,161], but it does not elucidate the exact mechanism at the basis of the increased neurotransmitter release. An enhanced neurotransmitter release efficiency, without signs of neuromuscular junction degeneration, was observed also in a Drosophila HD model expressing the full-length Huntingtin carrying a polyQ expansion of 128 glutamines. This increased neuromuscular junction activity preceded the formation of nuclear Huntingtin aggregates and the onset of HD symptoms and it was associated to an increased cytosolic Ca^2+^ concentration of the pre-synaptic elements at the neuromuscular junctions [162]. The possibility of nerve degeneration was ruled out also in a study carried out on levator auris longus muscle of 12–13 weeks old R6/2 mice, where no morphological alterations of the neuromuscular junctions, nor markers of denervation were found. Indeed, the mRNA levels of nAChRγ, SK3 and Scn5, which upregulate after denervation, were normal or downregulated in R6/2 muscle, in comparison with age-matched control animals. In this context, the possibility of a nerve transmission impairment, which may be ascribed to a decreased amount of vesicles released per action potential, was proposed as a mechanism that may compensate for the muscle hyperexcitability and contribute to motor impersistence [163]. An impaired synaptic vesicle exocytosis was found also in the diaphragm of three months old BACHD mice. Although no morphological anomalies of the neuromuscular junctions were revealed, a reduction of the synaptic elements and alterations in the synaptic vesicle shape and size were reported. Interestingly, neuromuscular dysfunction was reported in the absence of protein aggregates and before the onset of typical HD symptoms [164]. 

At rest, membrane potential of muscle fibers is assured mainly by the activity of the muscle chloride channel, ClC-1, and the Kir2.1 potassium channel. Any alteration of the potassium and chloride conductance leads to membrane hyperexcitability and prolonged repolarization times after nerve stimulation with consequent uncontrolled muscle contractions and rigidity, all symptoms that characterize the advanced phases of HD in human and in the animal models of HD. In the flexor digitorum brevis and interosseous muscles of R6/2 mice at late stage of the disease (12 weeks of age), the reduced expression of the ClC-1 channel was due to splicing aberration and downregulation of its mRNA levels; analogously, the mRNA levels of Kir2.1, Kv1.5 and Kv3.4 K^+^ channels were significantly reduced, in comparison with their wild-type littermates. The observed alterations of many parameters characterizing the action potentials in the flexor digitorum brevis and interosseous muscles and the consequent aberrations in their contractile functions may probably be ascribed to the transcriptional changes involving the Cl^-^ and K^+^ channels rather than to a denervation process [165,166]. On the other hand, many other studies indicate that nerve degeneration may be a primary defect underlying muscle wasting. A remarkable reduction of the axon and axoplasm diameter of myelinated neurons and increase of degenerating myelinated fibers, without alterations in myelin sheath, were observed in pre-symptomatic R6/2 mice compared to age-matched control animals. This suggests that axonal degeneration could be a primary cause of HD symptoms [167]. In the sternomastoid and in the tibialis anterior muscles from 12 months old male BACHD mice, an overall morphological aberration of the motor units, including motoneurons, axons, neuromuscular junction and muscle fibers was observed, indicating that both, the cervical and the lumbar spinal cord segments, that innervate different groups of muscles, were affected. In both spinal cord segments, there was a reduction in motoneuron number and size and neuromuscular fragmentation, in comparison with age-matched control animals. The reduced number of motoneurons could be ascribed to the activation of apoptotic processes, as confirmed by caspase-3 overexpression. Although a reduction of the axon and axoplasm diameters was measured, no changes in the number of axons in the sciatic nerve were observed. These aberrations were correlated to a marked muscle atrophy and fiber-type switching, from type IIb to type IIx in tibialis anterior and from IIx to IIa in sternomastoid muscles, respectively. However, no alterations of the transcriptional levels of genes, whose expression changes upon denervation, such as Chrna1, chrng, Gdf5 and Runx1, nor changes of the synaptotagmin protein levels were observed [79,168]. In contrast, an analysis performed on different types of skeletal muscle, with a different composition of muscle fiber types, such as extensor digitorum longus (fast-type muscle), soleus (slow-type muscle) and tibialis anterior or gastrocnemius/plantaris complex (mixed fiber compositions), revealed a remarkable upregulation of Ampd3, a gene related to muscle disuse and muscle denervation [169]. A significant loss of motor units was revealed by analyzing the extensor digitorum longus muscle from R6/2 mice at 12 weeks of age, a phenomenon that became more pronounced at more advanced stages of the disease, suggesting that the widely diffused muscle atrophy observed in the animal model might be driven by denervation [68,73]. In support to this hypothesis, it was reported an upregulation of the Histone deacetylase 4 (HDAC4) gene in tibialis anterior, extensor digitorum longus and gastrocnemius/plantaris complex muscles from R6/2 and HdhQ150 knock-in mice. It should be noted that HDAC4 links neural activity to the muscle remodeling process and inhibition of the HDAC4 activity prevented muscle atrophy induced by denervation while promoting re-innervation program [73].

## 10. Therapies for HD Treatment 

A vast number of drugs is currently under study for treatment of different symptoms of HD, as reviewed in many worthy articles [170,171]. Inhibition of the myostatin/activin A signaling, by injecting in R6/2 mice a soluble inactive form of the activin type IIB receptor, which competes with the natural receptor for circulating agonists, restored the normal fiber diameter, muscular mass, contractile functions and number of motor units at the wild-type levels [68]. Myostatin is a secreted growth factor, belonging to the transforming growth factor β super family that modulates muscle size, through its binding to the activin type IIB receptor, and deletion of its gene leads to muscular hypertrophy [172,173]. Interestingly, the rescue of the normal phenotype occurred without reducing nuclear aggregates, but rather increasing the percentage of nuclei containing Huntingtin aggregates that were increased in size [68]. Probably, inhibition of the myostatin/activin A signaling re-activated some transcriptional patterns, compensating for transcriptional dysfunctions linked to the presence of mutant Huntingtin and preventing the continuous replenishing of damaged muscle fibers with new centrally nucleated satellite cells, in which the formation of Huntingtin aggregates has not yet occurred [68]. This supports the view that the soluble forms of the mutant Huntingtin would be more harmful than its aggregates.

Many drugs targeting oxidative stress and mitochondrial dysfunctions have shown to ameliorate HD symptomatology [174]. For example, treating R6/2 mice with bezafibrate, an agonist of PPARs, restored the mRNA levels of PGC-1α. This brought about various beneficial effects, such as an improvement of mitochondrial shape and alignment, an appropriate redistribution of type I and type II muscle fibers, an increased muscular strength and performances and an extension of the life span [92]. Coenzyme Q10, a component of the electron transport chain, raised an initial enthusiasm for its ability to alleviate muscle dysfunction by promoting aerobic respiration and scavenging toxic reactive oxidative species [175,176,177]. Unfortunately, further large-scale studies, carried out on HD patients [178] and HD mouse models [179], demonstrated that coenzyme Q10 does not provide significant benefits in HD. Furthermore, creatine and eicosapentaenoic acid improved muscle performances by reducing mitochondrial damages in HD patients, but failed to rescue neuromuscular and cognitive functions [180,181]. Molecules aimed at reducing inflammation attracted a lot of interest, such as Laquinimod that plays an immunomodulatory role [182] and improved motor function and striatal neuropathology in R6/2 HD mice [183]. Antibody blockage of Semaphorin 4D, a transmembrane signaling protein that acts as immunomodulatory agent, displayed beneficial effects on YAC128 HD mice [184]. Molecules that inhibit TNF-α signaling have been also considered for their anti-inflammatory effects, but they exhibited limited ability to alleviate HD symptoms [185]. Currently, only two drugs achieved approval for treatment of HD, namely, the Tetrabenazine and its deuterated variant Deutetrabenazine, which showed better tolerability. These two molecules block the dopamine pathway by inhibiting vesicular monoamine transporter (VMAT) type 2. However, the therapeutic relevance of these two drugs is limited to the treatment of chorea and other motor dysfunction, as they are not able to block or slow down the HD progression [186,187,188,189]. It is now clear that the only therapeutic strategies that could really block the HD progression must be aimed at eliminating or at least reducing the levels of mutant Huntingtin. Indeed, drugs that facilitate the removal of the mutant Huntingtin misfolded variants, by promoting proteasome activity and macroautophagy, showed beneficial effects [190]. The efficacy of a vaccination based on the immunogenicity of three non-overlapping peptides identified within the amino acid sequence of the Huntingtin exon 1 was tested in preliminary studies [191,192]. Reduction of mutant Huntingtin aggregation and toxicity was also observed in cell culture and animal models of HD treated with different recombinant antibodies directed against Huntingtin [185]. Inhibition of Huntingtin mRNA translation has been successfully obtained with non-coding RNAs, such as small interfering RNAs, short hairpin RNAs, microRNAs, as well as with small molecules, such as coumarin derivatives [193]. Further therapeutic approaches likely to be developed in the next future are based on the CRISPR/Cas9 gene editing that could be employed to excise the abnormal CAG repeats or to introduce missense mutations, in order to render mutant Huntingtin innocuous [193]. At this time, the most promising therapeutic application is based on antisense oligonucleotides (ASOs) that are short single-stranded synthetic DNA oligomers composed of 8–50 nucleotides that bind mRNAs through Watson–Crick base pairing. The DNA-RNA double strands meet three different fates: degradation by endogenous enzymes, such as ribonuclease H, inhibition of mRNA translation or modulation of mRNA splicing [194]. For HD treatment, two categories of ASOs were developed, one that targets both, wild-type and mutant Huntingtin mRNAs, and the other that selectively targets mutant Huntingtin mRNA. This latter offers the advantage of maintaining adequate levels of wild-type Huntingtin that are needed for many cellular functions [193,195]. Therapeutic agents targeting RNA or DNA must be delivered to the cells, a particularly challenging task, especially for the central nervous system that can be reached by crossing the blood-brain barrier. From this point of view, skeletal muscle represents a more accessible tissue that can be targeted by any kind of drugs more easily. As a matter of fact, different studies demonstrated that rescue of the muscular function may mitigate the effects of a systemic disease, such as HD, even if the underlying molecular mechanisms of the disease are not targeted [68,92]. This can be explained considering that muscle releases myokines that produce beneficial effects for many organs, including the brain, and therefore, its correct function may have positive widespread repercussions.

## 11. Conclusions

The high expression levels of Huntingtin in skeletal muscle render this tissue heavily involved in the pathophysiology of the HD. The occurring of soluble and non-soluble oligomeric forms of mutant Huntingtin causes mitochondrial dysfunction, related to loss of energy homeostasis, oxidative stress, inflammation and apoptosis. In addition, it probably impairs the efficiency of the protein quality control systems, such as ubiquitin-proteasome system and macroautophagy, further exacerbating damages driven by the accumulation of mutated Huntingtin aggregates (see Figure 1). Mutant Huntingtin promotes these pathologic events by triggering a profound alteration of the transcriptome, which has widespread repercussions. Moreover, it is very likely that the mutant Huntingtin establishes aberrant interactions with mitochondria, autophagosome and proteasome, altering their physiologic functions, as demonstrated in other tissues. Very few studies have been focused on defects of the protein quality control systems in HD skeletal muscle, but there are many clues suggesting that impairment of these processes may play an important role in muscle dysfunction, so that it would be very useful to investigate this aspect further. In the light of these considerations, preventing the accumulation of misfolded forms of mutant Huntingtin, by stimulating macroautophagy and/or proteasome-driven protein degradation, could represent a rational therapeutic approach. 

Interestingly, many pathologic events occurring in HD skeletal muscle, such as mitochondrial dysfunction, oxidative stress, inflammation and reduced efficiency of the protein quality control systems, also characterize the natural aging process, leading to loss of muscle mass and strength, called sarcopenia. From this point of view, the HD muscle phenotype resembles that of a precociously aged muscle. This suggests that some therapeutic interventions that are beneficial for sarcopenia can be introduced in the treatment of HD.

There are two still open issues: the first one is whether muscle dysfunction is a primary defect, or a consequence of nerve degeneration. The second one is the reason for which the detrimental effects driven by the expression of mutant Huntingtin are more severe for some organs than others, although Huntingtin and its pathological variants are expressed at similar levels in many organs, such as liver, lung, kidney, testis, beside brain and skeletal muscle. Further studies are needed to obtain a deeper comprehension of these aspects.

## Figures and Tables

**Table 1:** Animal models for HD mentioned in the review.

Name	Type	DNA Construct	CAG Repeats Length	AGE of Onset	Mutant HTT Expression*	Reference
R6/2 mouse	Transgenic	Promoter and exon 1 of human Htt gene	144	9–11 weeks (juvenile HD)	75%	[43]
R6/1 mouse	Transgenic	Promoter and exon 1 of human Htt gene	116	4–5 months	30%	[43]
NLS-N171-82Q mouse	Transgenic	Mouse prion promoter and human sequence for the first 171 N-terminal amino acids of Htt	82	9–11 weeks (juvenile HD)	10–20%	[44,45,46,47]
HdhQ_n_ mouse	Knock-in	Knock-in of variable number of CAG repeats into mouse Htt locus	92–200	Depending on the length of the CAG repeats	100%	[40,41,42,48,49]
BACHD mouse	Transgenic	Full-length human Htt gene under human Htt promoter	97	Slow disease progression and normal life span	100%	[50,51]
YACQ_n_ mouse	Transgenic	Full-length human Htt gene with variable number of CAG repeats under human Htt promoter	48–128	Depending on the length of the CAG repeats	30–100%	[52,53,54,55]
Mini pigs	Transgenic	Human promoter and human sequence for the first 548 N-terminal amino acids of Htt	124	Slow progression of the disease	100%	[56]

Q_n_: variable (*n*) glutamine repeats; BAC: bacterial artificial chromosome; YAC: yeast artificial chromosome vector system; Htt: Huntingtin. *relative to endogenous level.
